# Increased effort during partial ventilatory support is not associated with lung damage in experimental acute lung injury

**DOI:** 10.1186/s40635-019-0272-z

**Published:** 2019-11-05

**Authors:** Dietrich Henzler, Alf Schmidt, Zhaolin Xu, Nada Ismaiel, Haibo Zhang, Arthur S. Slutsky, Paolo Pelosi

**Affiliations:** 1 Department of Anesthesiology, Dalhousie University, Halifax, Nova Scotia Canada; 2 Department of Physiology and Biophysics, University of Toronto, Toronto, ON Canada; 3 Anesthesia and Surgical Intensive Care, Ruhr-University Bochum, Bochum, Germany; 4 Department of Anesthesia, Surgical Intensive Care, Emergency and Pain Medicine, Ruhr-University Bochum, Klinikum Herford, Schwarzenmoorstr. 70, 32049 Herford, Germany; 5 Department of Pathology, Dalhousie University , Halifax, NS Canada; 6 Department of Anesthesia, University of Toronto, Toronto, ON Canada; 7 Interdepartmental Division of Critical Care Medicine, University of Toronto, Toronto, Canada; 8 Keenan Research Center at the Li Ka Shing Knowledge Institute of St. Michael’s Hospital, Toronto, ON Canada; 9 Department of Surgical Sciences and Integrated Diagnostics, San Martino Policlinico Hospital - IRCCS for Oncology, University of Genoa, Genoa, Italy

**Keywords:** Acute lung injury, ALI, Acute respiratory distress syndrome, ARDS, Ventilator-associated lung injury, VALI, Mechanical ventilation, Assisted spontaneous breathing

## Abstract

**Background:**

An on-going debate exists as to whether partial ventilatory support is lung protective in an acute phase of ARDS. So far, the effects of different respiratory efforts on the development of ventilator-associated lung injury (VALI) have been poorly understood.

To test the hypothesis whether respiratory effort itself promotes VALI, acute lung injury (ALI) was induced in 48 Sprague Dawley rats by hydrochloric acid aspiration model. Hemodynamics, gas-exchange, and respiratory mechanics were measured after 4 h of ventilation in pressure control (PC), assist-control (AC), or pressure support with 100% (PS100), 60% (PS60), or 20% (PS20) of the driving pressure during PC. VALI was assessed by histological analysis and biological markers.

**Results:**

ALI was characterized by a decrease in PaO_2_/FiO_2_ from 447 ± 75 to 235 ± 90 mmHg (*p* < 0.001) and dynamic respiratory compliance from 0.53 ± 0.2 to 0.28 ± 0.1 ml/cmH_2_O (*p* < 0.001). There were no differences in hemodynamics or respiratory function among groups at baseline or after 4 h of ventilation. The reduction of mechanical pressure support was associated with a compensatory increase in an inspiratory effort such that peak inspiratory transpulmonary pressures were equal in all groups. The diffuse alveolar damage score showed significant lung injury but was similar among groups. Pro- and anti-inflammatory proteins in the bronchial fluid were comparable among groups.

**Conclusions:**

In experimental ALI in rodents, the respiratory effort was increased by reducing the pressure support during partial ventilatory support. In the presence of a constant peak inspiratory transpulmonary pressure, an increased respiratory effort was not associated with worsening ventilator-associated lung injury measured by histologic score and biologic markers.

## Background

Ventilator-associated lung injury (VALI) is a complication of mechanical ventilation. Tidal volume, airway pressure, and cyclic opening and closing of alveolar lung regions have been shown to impact the degree of VALI. A lung-protective ventilation strategy with reduced tidal volume [1], limited inspiratory plateau and driving pressure [2], and positive end-expiratory pressure (PEEP) [3] has been shown to improve outcomes in patients with acute respiratory distress syndrome (ARDS).

Controlled modes of mechanical ventilation that deliver full ventilatory support have been used to completely offload the work of breathing and thereby “rest the respiratory muscles.” In a recent clinical trial of patients with moderate to severe ARDS, abolishing spontaneous breathing activity with muscle paralysis during the first 48 h of treatment improved survival [4]. On the other hand, partial ventilatory support allows spontaneous breathing efforts during mechanical ventilation and preserves respiratory muscle function [5]. Partial ventilatory support, traditionally reserved for use in weaning, is now often used in all phases of mechanical ventilation [6, 7], although the potential of increased respiratory effort to the dependent lung to contribute to VILI is a matter of debate [8].

Preserved spontaneous breathing during partial ventilatory support is potentially protective because it may improve the homogeneity of ventilation and ventilation-perfusion distributions. Several experimental studies have investigated the role of spontaneous breathing on VALI. If spontaneous breathing was associated with a decrease in peak inspiratory transpulmonary pressure (*P*_TPi_), the development of VALI was attenuated with both higher [9] or decreased [10] inspiratory effort. On the other hand, the increased respiratory drive has been demonstrated to cause potentially injurious swings in transpulmonary pressure, so-called patient self-inflicted lung injury (P-SILI) [11] with a concomitant increase in *P*_TPi_ that presumably augmented VALI [12].

Thus, it seems that the effect of spontaneous breathing on VALI is mainly related to peak *P*_TPi_. However, the role of inspiratory effort independent of *P*_TPi_ was not investigated. We hypothesized that increased inspiratory effort, maintaining peak *P*_TPi_ constant, is associated with VALI.

## Materials and methods

Sixty-three male Sprague Dawley rats (456 ± 70 g, Charles River Laboratories, Wilmington, MA) were anesthetized with pentobarbital 55 mg/kg intraperitoneally and instrumented as described previously [13] (for a more detailed description see Additional file 1). Briefly, animals were tracheotomized with a 14 G tube and mechanically ventilated (EVITA4, Draeger Medical Canada Inc., Richmond, ON, Canada) with inspired oxygen fraction (FiO_2_) of 0.4. Twenty gauge catheters were inserted into the carotid artery and jugular vein for blood pressure monitoring and blood gas analysis (ABL510 + OSM3, Radiometer Copenhagen, Denmark). The FiO_2_ was set to 1.0 2 min before obtaining blood samples for gas analysis and set back to 0.4 after each measurement. The femoral artery was cannulated with a thermocouple probe (ADInstruments Inc., Colorado Springs, CO, USA) for cardiac output measurements. Sedation was maintained by infusion of ketamine 20 mcg/kg/min intravenously.

### Measurements

Cardiac output was measured by transcardio-pulmonary temperature dilution of 0.5 ml of saline solution (LabChart 6.0, ADInstruments). Gas flow and airway pressures (*P*_AW_) were measured proximal to the endotracheal tube by a heated pneumotachograph (Hans Rudolph Inc., Shawnee, KS). Esophageal pressure (*P*_ES_) was measured via a water-filled 20 G catheter with multiple perforations inserted into the esophagus. Dynamic compliance of the respiratory system was defined as tidal volume (*V*_*T*_) divided by the pressure difference between end-inspiratory and end-expiratory airway pressure. The maximum difference between end-inspiratory airway pressure and *P*_ES_ was defined as peak inspiratory transpulmonary pressure (*P*_TPi_). Respiratory effort was assessed by calculation of pressure-time-product (PTP) and work of breathing (WOB), which were calculated separately for total (WOB_T_), ventilator (WOB_V_), and respiratory muscles (WOB_RM_) from the recorded flow and *P*_ES_ tracings according to standard formulae (see Additional file 1). A data collection system was used (PowerLab, ADInstruments).

### Experimental protocol

Initially, sedated animals were paralyzed with additional pancuronium 0.5 mg/kg/h intravenously and ventilated using pressure-controlled ventilation. The level of inspiratory driving pressure (ΔP_AW_) was set to achieve *V*_*T*_ of 8 ml/kg, and the mechanical respiratory rate was set to maintain a PaCO_2_ < 60 mmHg, while avoiding flow at end-expiration. The positive end-expiratory pressure (PEEP) was set to 5 cmH_2_O and inspiration-expiration ratio set to 1:1. Lung injury was induced by intra-tracheal instillation of 0.5 ml of 0.2 m HCl as described previously [13]. Physiologic measurements were performed at baseline (BL) and 1 h after induction of lung injury (ALI-BL) (Additional file 1: Figure S1). A recruitment maneuver was performed by increasing PEEP to 10 cmH_2_O for 2 min, and animals were afterwards randomized to one of the following 5 groups:

1. *PC*: Pressure control with neuromuscular blockade and unchanged ventilatory settings.
2. *AC*: Assist-control mode without paralysis and the pressure controlled mechanical breaths set as before.
3. *PS100*: Spontaneous breathing with pressure support (PS) set equal to 100% of the previous Δ*P*_AW_ (value required to obtain a *V*_*T*_ of 8 ml/kg during pressure control).
4. *PS60*: Spontaneous breathing with PS set equal to 60% of the previous Δ*P*_AW_.
5. *PS20*: Spontaneous breathing with PS set equal to 20% of the previous Δ*P*_AW_.

After randomization, neuromuscular blockers were discontinued in groups 2–5. Animals remained in these settings for 4 h. Only if needed, the Δ*P*_AW_ was adjusted to keep the *V*_*T*_ < 10 ml/kg in case of changing respiratory system compliance. In PS, the respiratory rate was self-adjusted by the animals. Physiologic measurements were taken hourly with the final measurement after 4 h or the last one before death (ALI-End) (Additional file 1: Figure S1). Only animals receiving at least 2 h of the study ventilation regimen were included in the analysis. Arterial and venous blood samples were taken for cytokine analysis, then the animals were killed and the lungs dissected for histologic analysis as described previously [13]. The right middle lobe was weighed and dried for 48 h at 37 °C for analysis of wet-to-dry ratio.

### Histopathology and cytokine analysis

After formalin fixation, the lungs were embedded in paraffin, cut, and stained with hematoxylin eosin. A lung pathologist blinded to the experimental group (Z.X.) graded the degree of diffuse alveolar damage (DAD) [13] (Additional file 1: Table S2).

Blood samples were immediately centrifuged and supernatant plasma stored at − 80°. Broncho-alveolar lavage fluid (BALF), and plasma samples were analyzed by multiple enzyme-linked immune assay (Luminex® technology, Panomics Inc., Fremont, CA) for tumor necrosis factor-α (TNF-α); interleukin (IL)-1β, IL6, and IL10; intracellular adhesion molecule (ICAM1); macrophage inflammatory protein (MIP1α); and KC (CXCL1 chemokine). Regulated upon Activation Normal T-cell Expressed and Secreted (RANTES) and Monocyte Chemotactic Protein (MCP1). Standard dilution curves were constructed to calculate concentrations in pg/ml.

### Statistical analysis

Data were tested for normality by Kolmogorov-Smirnov test and also empirically based on visual inspection of Q-Q plots. Parametric data are presented as mean ± SD, while non-parametric data are expressed as median (interquartile range). Since sample size calculation is difficult, we assumed an effect size similar to previously published experiments [10–13] giving 8–10 animals per group. The sample size was based on pilot studies and on our past experience with ventilator strategies in small animals. We tested the hypothesis that increased WOB_RM_ would increase DAD during assisted ventilation. Using data from previous experiments and a calculated effect size of 0.531, a sample size of 10 animals per group would provide the appropriate power (1-*β* = 0.82) to identify significant (*α* = 0.05) differences (G*Power 3.1.9.3, Duesseldorf, Germany).

Included for analysis were only animals that had completed at least 2 h of study ventilation after ALI-BL measurement. Baseline measurements were not included in the analysis, but are shown for informative purpose only. For parametric data, physiologic measurements were analyzed by a mixed-model ANOVA for repeated measurements with Wilk’s lambda indicating significance for between-group and within-group differences. If significant, post hoc comparison using SNK correction was used to analyze between-group differences. Repeated measurements were analyzed by paired *t* test and correlations by Spearman’s rho. Cytokines and W/D ratios were analyzed by one-way ANOVA, followed by post hoc comparison using SNK correction. Non-parametric data was analyzed accordingly by Kruskal-Wallis followed by a Mann-Whitney *U* test as appropriate. Categorical data were analyzed by chi-square (Statistical Package for the Social Sciences 15.0, SPSS Inc., Chicago, Ill, USA).

## Results

All data except DAD score were normally distributed. There were no differences in hemodynamics or respiratory mechanics among groups at baseline. Intra-tracheal HCl caused similar hemodynamic and respiratory function compromise. Fifteen experiments had to be terminated early for various reasons, i.e., inadvertent severe lung damage, problems with instrumentation, handling, malfunction of equipment, drug application, hemodynamic compromise of other reason, and protocol violation. These experiments were stopped, and no analyses of cytokines or histology were performed, leaving 48 animals for analysis. Averaging data for all groups, the mean arterial pressure decreased from 144 ± 23 at BL to 119 ± 27 mmHg at ALI-BL (*p* < 0.001), although cardiac output remained stable at 122 ± 30 and 121 ± 34 ml/min (*p* = 0.34), respectively. Lung injury was characterized by a decrease in PaO_2_/FiO_2_ from 447 ± 75 to 235 ± 90 mmHg (*p* < 0.001) and in dynamic compliance from 0.53 ± 0.2 to 0.28 ± 0.1 ml/cmH_2_O (*p* < 0.001). At ALI-BL, the respiratory mechanics’ data were comparable among groups (Table 1).

**Table 1:** Respiratory mechanics

		PC (*n* = 9)	AC (*n* = 10)	PS100 (*n* = 9)	PS60 (*n* = 10)	PS20 (*n* = 10)	Main effect	
							Within group	Between group
*P*_AW_ [cmH_2_O]	BL	15 ± 3	15 ± 4	13 ± 2	14 ± 3	13 ± 1		
	ALI-BL	22 ± 4	22 ± 4	18 ± 3	20 ± 4	21 ± 1		
	ALI-End	26 ± 6#*	25 ± 7	19 ± 7	15 ± 5*	9 ± 3#*	0.060	0.000
*P*_TP_insp [cmH_2_O]	BL	16 ± 4	15 ± 4	13 ± 4	14 ± 4	13 ± 5		
	ALI-BL	22 ± 5	23 ± 4	20 ± 6	19 ± 6	19 ± 4		
	ALI-End	25 ± 7	26 ± 7	24 ± 9	23 ± 6	23 ± 8	0.006	0.987
*P*_TP_exp [cmH_2_O]	BL	5 ± 3	4 ± 2	4 ± 3	6 ± 2	4 ± 4		
	ALI-BL	4 ± 3	5 ± 2	6 ± 5	4 ± 3	3 ± 3		
	ALI-End	5 ± 4	5 ± 4	6 ± 4	4 ± 2	3 ± 6	0.790	0.337
*V*_*T*_ [ml/kg BW]	BL	9.6 ± 1.2	8.7 ± 0.8	8.7 ± 0.6	8.8 ± 1	8.6 ± 0.9		
	ALI-BL	8.4 ± 1.6	9.4 ± 2.0	8.9 ± 2.8	11 ± 4	8.6 ± 1.9		
	ALI-End	9.0 ± 1.2	9.0 ± 1.2	9.1 ± 1.0	10 ± 3	8.9 ± 1.2	0.919	0.853
RR [min^−1^]	BL	77 ± 18	81 ± 15	84 ± 8	87 ± 7	85 ± 9		
	ALI-BL	75 ± 17	82 ± 14	85 ± 11	92 ± 6	84 ± 11		
	ALI-End	86 ± 16#	94 ± 17#	71 ± 31	77 ± 17#	80 ± 20	0.213	0.011
*V*_*E*_ [ml/min]	BL	334 ± 115	328 ± 68	348 ± 42	353 ± 56	339 ± 48		
	ALI-BL	323 ± 60	346 ± 86	364 ± 82	403 ± 91	352 ± 58		
	ALI-End	318 ± 70	411 ± 117	320 ± 115	396 ± 123	330 ± 130	0.662	0.345

*BL* baseline measurement on pressure controlled ventilation, *ALI-BL* measurement on pressure-controlled ventilation after induction of lung injury, *ALI-End* last measurement after 2–4 h of experimental ventilation mode, *P*_*AW*_ peak airway pressure, *P*_*TP*_*i* peak inspiratory transpulmonary pressure, *V*_*T*_ tidal volume, *RR* respiratory rate, *V*_*E*_ minute ventilation, *p < 0.05 vs. other groups #*p* < 0.05 vs. ALI-BL

After randomization, *V*_*T*_ remained similar among groups (Table 1), but Δ*P*_AW_ had to be increased in PC and decreased in PS100 to maintain the target *V*_*T*_. Minute ventilation remained constant despite the reduction of ventilatory support. *P*_TPi_ was similar among groups, independent from the level of ventilatory support (Table 1, Additional file 1: Figure S2).

The level of ventilatory support did not significantly affect hemodynamics. The mean arterial pressure, but not cardiac output, decreased similarly in all groups over time (Table 2). However, animals in PS20 exhibited more episodes of hemodynamic destabilization, leading to the lowest rate of animals achieving 240 min of ventilation (PC 44%, AC 70%, PS100 55%, PS60 60%, and PS20 30%; *p* = 0.68). In prematurely deceased animals, the time between ALI-BL and death was on average 211 ± 35 min, which was not significantly different among groups (Additional file 1: Table S1).

**Table 2:** Hemodynamics and gas exchange

		PC (*n* = 9)	AC (*n* = 10)	PS100 (*n* = 9)	PS60 (*n* = 10)	PS20 (*n* = 10)	Main effect	
							Within group	Between group
HR [min^−1^]	BL	420 ± 33	394 ± 43	388 ± 90	405 ± 32	370 ± 36		
	ALI-BL	377 ± 65	382 ± 45	374 ± 53	375 ± 65	410 ± 41		
	ALI-End	357 ± 96	324 ± 67#	353 ± 64	342 ± 39	375 ± 42#	0.005	0.830
MAP [mmHg]	BL	139 ± 26	134 ± 32	152 ± 15	150 ± 16	147 ± 23		
	ALI-BL	101 ± 37	120 ± 24	124 ± 22	125 ± 23	124 ± 24		
	ALI-End	47 ± 40#	74 ± 44#	80 ± 16#	78 ± 26#	72 ± 35#	0.000	0.943
CO [ml/min]	BL	116 ± 44	107 ± 27	128 ± 24	130 ± 27	124 ± 26		
	ALI-BL	92 ± 29	103 ± 17	147 ± 30	129 ± 18	146 ± 41		
	ALI-End	128 ± 47	91 ± 52	130 ± 29	132 ± 29	113 ± 42	0.575	0.139
pHa	BL	7.26 ± 0.09	7.26 ± 0.06	7.25 ± 0.08	7.25 ± 0.07	7.28 ± 0.05		
	ALI-BL	7.22 ± 0.11	7.23 ± 0.07	7.22 ± 0.06	7.24 ± 0.07	7.22 ± 0.05		
	ALI-End	7.07 ± 0.12#	7.08 ± 0.15#	7.04 ± 0.19#	7.13 ± 0.15#	7.04 ± 0.09#	0.000	0.763
PaO_2_ [torr]	BL	445 ± 78	440 ± 76	451 ± 85	448 ± 84	450 ± 67		
	ALI-BL	204 ± 119	234 ± 105	271 ± 91	243 ± 73	240 ± 62		
	ALI-End	92 ± 50#	118 ± 108#	166 ± 126#	106 ± 67#	83 ± 64#	0.000	0.873
PaCO_2_ [torr]	BL	59 ± 19	59 ± 10	59 ± 14	59 ± 10	57 ± 11		
	ALI-BL	60 ± 14	57 ± 14	61 ± 13	61 ± 14	65 ± 11		
	ALI-End	74 ± 27	68 ± 24	78 ± 19#	79 ± 26#	87 ± 20#	0.000	0.734

*BL* baseline measurement on pressure controlled ventilation, *ALI-BL* measurement on pressure-controlled ventilation after induction of lung injury. Blood gas analyses performed with FiO_2_ = 1.0. *ALI-End* last measurement after 2–4 h of experimental ventilation mode, *HR* heart rate, *MAP* mean arterial pressure, *CO* cardiac output, *p < 0.05 vs. other groups #*p* < 0.05 vs. ALI-BL

After the establishment of lung injury, oxygenation continued to deteriorate similarly in all groups from ALI-BL to ALI-End (Table 2). PaCO_2_ inversely correlated to reduced ventilatory support and tended to be highest in PS20 (n.s.). The increase in PaCO_2_ was significant only for pressure support groups.

**Fig. 1:**
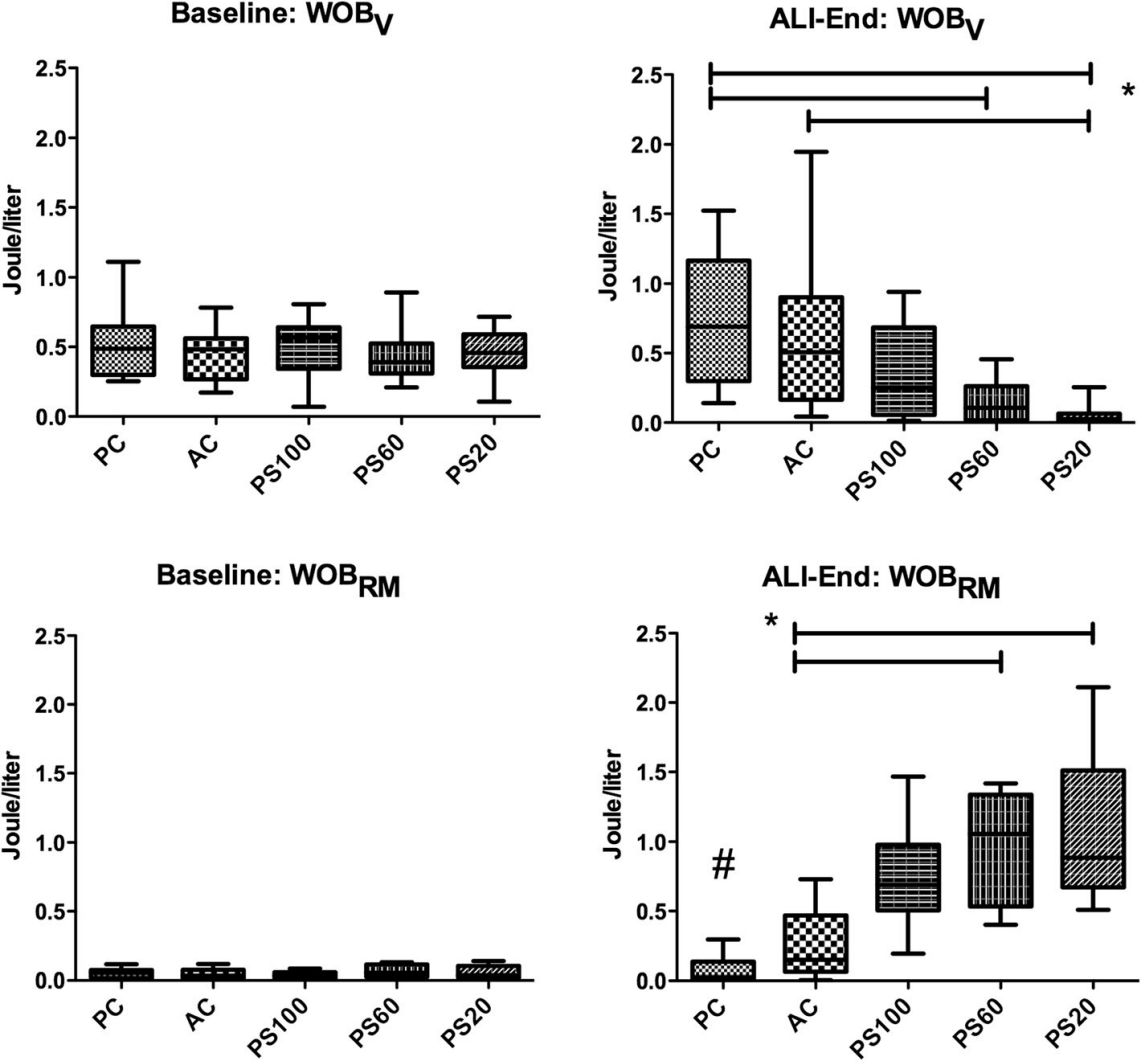
Work of breathing exerted by the animal (WOB_RM_) at baseline and after 4 h (ALI-End) of ventilation with pressure control (PC), assist control (AC), or pressure support ventilation with 100%(PS100), 60%(PS60), or 20%(PS20) of previous pressure control level after establishment of ALI.*p < 0.05 vs. indicator line; ^#^*p* < 0.05 vs. all other groups

As expected, the establishment of ALI was associated with an increase in the average WOB_T_ from 0.46 ± 0.2 J/l at BL to 0.95 ± 0.47 J/l at ALI-BL (*p* < 0.001). The calculated WOB_V_ was highest in PC and decreased with ventilatory support to a minimum with PS20. The WOB_RM_ was lowest in PC and increased with reduced ventilatory support (Fig. 1, Additional file 1: Table S3). The pressure-time product behaved similar to WOB_RM_; both parameters correlated significantly (*r* = 0.742, *p* < 0.001). Across all groups, the higher the work performed by the ventilator (WOB_V_), the lower the mean arterial pressure (*r* = − 0.389, *p* = 0.007) (Additional file 1: Figure S3).

**Fig. 2:**
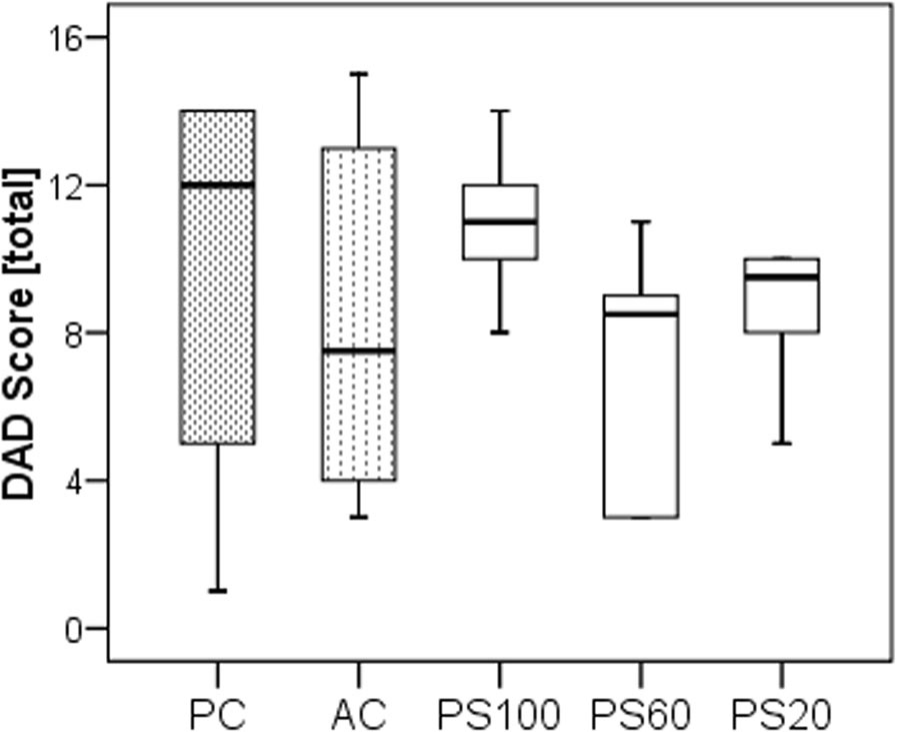
Diffuse alveolar damage (DAD) total score after 4 h of ventilation in pressure control (PC), assist control (AC), or pressure support ventilation with 100%(PS100), 60%(PS60), or 20%(PS20) of previous pressure control level. Differences among groups were not statistically significant (*p* = 0.097)

There were no significant differences in wet-to-dry ratios among groups (Additional file 1: Figure S3). The wet-to-dry ratio decreased with the higher pressure-time product (*r* = − 0.305, *p* = 0.037). Lung damage was mainly related to the epithelium, neutrophil infiltration, and edema, but not hemorrhage. The level of ventilatory support did not affect the total alveolar damage score (Fig. 2) or its components (Additional file 1: Table S4). The alveolar damage score correlated inversely with pressure-time product (*r* = − 0.469, *p* = 0.049) (Additional file 1: Figure S6).

**Fig. 3:**
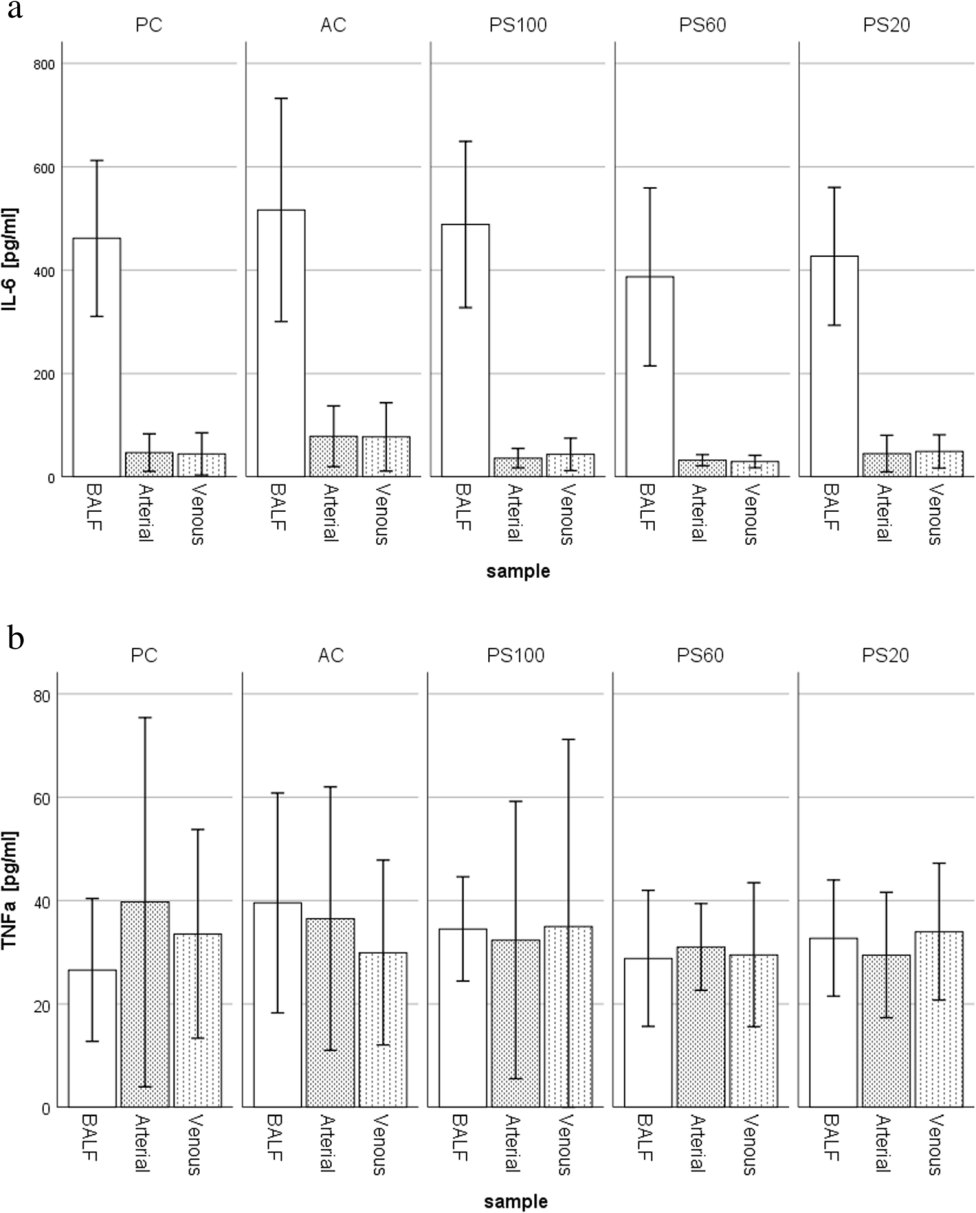
Measurement of cytokines in the bronchoalveolar fluid (BALF) and arterial (Art) and venous (Ven) samples. Data are presented as mean ± SD [pg/ml], upper panel (**a**): IL6. Measured levels were higher in the BALF than the plasma, but differences among groups were not statistically significant; lower panel (**b**): TNF-a. No significant differences were found between BALF and plasma or among groups

The concentrations of inflammatory markers were not different between arterial and venous blood samples and did not differ among the variated levels of ventilatory support (Additional file 1: Tables S5, S6, S7). IL-6 (Fig. 3a), KC, MIP1a, and MCP1 were higher in the BAL compared to plasma; IL1β, ICAM, and TNF-α (Fig. 3b) were equal in the BAL and plasma. RANTES was higher in plasma compared to BAL in all groups and IL-10 only in PS60 and PS20 groups.

## Discussion

In a rodent acid aspiration model of mild to moderate acute lung injury, we found that increased respiratory effort when generated such as to produce identical peak inspiratory transpulmonary pressure and tidal volume had no effect on gas exchange, histologic, and biologic markers of lung injury.

We tried to control for most known factors associated with the development of VALI. The lung injury model and dosage of HCl was chosen in order to achieve severe lung damage and keep the model stable during the experiment time. Protective ventilation was maintained both during controlled and partial ventilatory support ventilation, with a PEEP sufficient to prevent major regional lung collapse in small animals [13]. Sedation was administered continuously and equally in all groups. The duration of ventilation after induction of lung injury was at least 3 h making it reasonable to investigate the production of inflammatory proteins. Supposedly, previous studies investigating the influence of transpulmonary pressure on VALI resulted in different levels of *P*_TPI_. The innovative approach of our study was to investigate the effect of different levels of inspiratory effort (while keeping *P*_TPi_ constant) on VALI and the production of inflammatory proteins.

Despite varying levels of partial ventilatory support, the animals maintained *constant tidal volumes and minute ventilation*. The reduction of pressure support caused a compensatory increase in the respiratory effort to match previous *P*_TPi_ and preserve tidal volume. To achieve similar *P*_TPi_, the distribution of forces across the lungs must be different during partial ventilatory support: the alveolar pressure is lower and the pleural pressure more negative with progressive reduction in pressure support, which could impact hemodynamics. Consequently, the decrease of partial ventilatory support was associated with an increase of WOB_RM_ and pressure-time product.

Our data suggest that, during partial ventilatory support, *P*_TPi_ is more important than respiratory effort “per se” in generating VALI, suggesting that the major factor causing volutrauma is the degree of lung stretch [14, 15] .

Previous experimental studies showed a possible protective effect of hypercapnia on the development of VALI [16], although severe hypercapnia is associated with worse outcome in patients with ARDS if used as an indicator of the inability to sufficiently ventilate the failing lung [17]. In our study, all animals developed hypercapnia, but the increase was significant only for those ventilated in pressure support. IL-10 was the only mediator increasing in PS60 and PS20 groups, the modes with the highest PaCO_2_, demonstrating a possible, anti-inflammatory effect.

VALI is characterized by structural injury (alveolar damage score) and biochemical mediators. While cytokine mRNA expression is a sensitive measure, it is not necessarily followed by biologic injury. Conversely, the detection of active protein has the advantage of a definite effect, although proof of origin, i.e., the lung, may be problematic. The fact that some, but not all inflammatory markers (IL1β, TNF-α) had concentrations similar in BALF and plasma implies differences in alveolar secretion of biomarkers. Conversely, those cytokines that were increased in BALF, but not plasma (IL6, KC, MCP1), likely originate in the lung before spilling into the blood. The absence, presence, or amount of inspiratory effort did not affect lung-derived pro-inflammatory substances under the condition of a constant *P*_TPi._

Previous work by Yoshida et al. [10, 12] seems conflicting with our results. There are substantial differences in our study design that may explain some of the different findings. First, most important, we have controlled for the transpulmonary pressure which was equal in all groups rather than *P*_TP_ being an outcome of the ventilation strategy. The amount of alveolar damage was closely correlated with increased PTP. We have also used a more physiologic calculation of the total alveolar stretching pressure, as we calculated the pressures across the lung (*P*_TPi_) from real-time tracings. Yoshida et al. have used a fixed sum of *P*_PLAT_ + Δ*P*_es_, regardless whether it was becoming effective to the lungs. Second, we have used a more realistic ventilation strategy. Yoshida et al. have used chemical agents to increase respiratory drive and thereby added spontaneous breathing on top, increasing minute ventilation and PEEPi, a condition neither physiological nor clinically applied [12]. In another study Yoshida et al. used assist-control ventilation; however, from the printed curves, there is evidence of severe subject-ventilator dyssynchrony, another important mechanism of VALI [10]. In our study, pressure support was used to gradually increase respiratory effort while letting the subject maintain minute ventilation and controlling *P*_TPi_. Third, we used the most physiologic model of acid installation to induce lung injury [13]. The lavage model is prone to spontaneous improvement and very minimal influence on alveolar damage, while the extent of the actual lung injury is mainly depending on the ventilation settings. In contrast, the acid aspiration model creates characteristic inflammation and lung injury very similar to clinical situations. We did not specifically design our study to investigate the importance of spontaneous breathing on VALI. Although previous investigations suggested that strong spontaneous breathing in severe ARDS might augment lung injury [10], a large observational clinical study established an association of assisted ventilation with better outcome compared controlled mechanical ventilation and paralysis in patients from mild to severe ARDS [18].

We cannot exclude the possibility that increased effort during PS is associated with vascular injury, since DAD did not differentiate alveolar and perivascular hemorrhage. Previous studies have reported increased hemorrhage with the addition of respiratory effort, when rabbits were ventilated with tidal volumes of 7–9 ml/kg^12^. Additionally, DAD and hemorrhage were higher in AC with spontaneous breathing in severe lung injury and higher *P*_TPi_ [10]. However, in these studies, the work of breathing and pressure-time product were not measured. We found no difference in DAD and hemorrhage with increased respiratory effort.

Recent studies during volume control ventilation reported possible effects of increased mechanical power on lung injury [19, 20]. However, during assisted ventilation, the mechanical power to the lungs was delivered partly by the ventilator and partly by the inspiratory effort of the subject. In our study, the total mechanical power was similar at different ventilatory settings, suggesting that during assisted ventilation, it is mandatory to partition subject inspiratory effort.

Our study does have limitations that must be considered. First, we used an aspiration model in rats; this is a clinically relevant model of lung injury, but we cannot exclude the possibility that different injury models in different species may produce different results. We have used a model of lung injury, which may exhibit changes over time, but is relatively constant in these changes and the produced biological effect making it advantageous over other models [13]. Second, we investigated only pressure support and not other partial ventilatory support modalities. Third, those animals ventilated with a lower level of support had hypercapnic acidosis (HA) suggesting a higher grade of ventilation/perfusion mismatch. We cannot rule out the possibility that HA attenuated possible higher lung injury at lower levels of pressure support. Fourth, since we did not measure lung volume and recruitment, different values for strain and atelectrauma [21] could have influenced our results. However, if pressure across the lung and tidal inflation are constant, increasing activation of the respiratory muscles is not associated with increased lung damage. Fifth, our approach was a more dynamic and physiological one, rather than analyzing the spatial distribution of gas across the lungs. Even CT scanning is not able to differentiate between the collapsed lung and edematous lung in non-aerated areas. From histology in similar experiments [10], it is evident that non-aerated lung was mainly due to alveolar/interstitial edema and infiltrates. The selected level of PEEP (5 cmH_2_O) in small animals physiologically exerts equal effects to the lungs as higher levels in larger animals (i.e., likely equaling PEEP 10 cmH_2_O in rabbits and PEEP 15 cmH_2_O in dogs) or humans and thus appears appropriate to assure sufficient aeration. Further, the influence of edematous lung on Va/Q mismatch and atelectasis formation in the dependent lung is much less in rodents with lungs weighing a few grams only as compared to larger animals or humans. Sixth, we did not monitor neuromuscular blockage but assessed the absence or presence of spontaneous breathing by the esophageal tracings, a method that has been used by other investigators as well [10, 12]. Seventh, not all animals completed the planned 240 min of the experiment. Most experimental data in small animals come from shorter experiments, commonly being ≤ 2 h of mechanical ventilation [22, 23]. Considering the kinetics of cytokine development after injury, the measured plasma levels depend on the experimental setup. The lower rate of finishing animals in PC and PS20 might have been underestimated by a type II error. Although not powered to detect a difference in mortality among groups, our data suggest that the type of ventilation mode independently from the inspiratory effort might have an effect on survival.

## Conclusions

This study provides experimental evidence that in a clinically relevant animal model of acute lung injury, the degree of VALI is not correlated with increased respiratory effort per se, when tidal volume and end-inspiratory transpulmonary pressures are relatively constant across differing respiratory efforts. Our results suggest that in setting the ventilator during partial ventilatory support, P_TPi_ is more important than inspiratory effort to minimize VALI. In application to patient care, it has to be considered that these results were obtained with lung-protective settings and might not be true for different ventilator conditions. As a clinical consequence, monitoring of esophageal pressure may be useful to optimize ventilatory setting [24].

## Supplementary information

**Additional file 1:** Supplemental digital content

## Data Availability

The datasets used and/or analyzed during the current study are available from the corresponding author on reasonable request.

## Abbreviations

ALI: Acute lung injury
ARDS: Acute respiratory distress syndrome
BALF: Bronco-alveolar lavage fluid
C_DYN_: Dynamic compliance respiratory system
ICAM: Intracellular adhesion molecule
IL1β, -6, -10: Interleukin
IP: Intraperitoneal
IV: Intravenous
KC: CXCL1 chemokine
MCP1: Monocyte chemotactic protein
MIP1α: Macrophage inflammatory protein
*P*_AW_: Airway pressure
PC: Pressure control
*P*_ES_: Esophageal pressure
PS: Pressure support
PTP: Pressure time product
*P*_TPi_: Maximum inspiratory transpulmonary pressure
RANTES: Regulated upon activation normal T cell expressed and secreted
RR: Respiratory rate
TNF-α: Tumor necrosis factor α
*V*_*E*_: Minute ventilation
*V*_*T*_: Tidal volume
WOB_RM_: Work of breathing performed by respiratory muscles
WOB_T_: Total work of breathing
WOB_V_: Work of breathing performed by the ventilator
