# Supplementary material for: Increased effort during partial ventilatory support is not associated with lung damage in experimental acute lung injury
Source: Intensive Care Med Exp. 2019 Nov 5;7:60. doi: 10.1186/s40635-019-0272-z (PMC6831786; doi:10.1186/s40635-019-0272-z)

# Increased effort during partial ventilatory support is not associated with lung damage in experimental acute lung injury

Dietrich Henzler MD PhD [1,2,6,7]; Alf Schmidt[1]; Zhaolin Xu MD[3]; Nada Ismaiel MSc[2,4]; Haibo Zhang MD PhD[5]; Arthur S Slutsky MD[5]; Paolo Pelosi MD, FERS[6]

[1]Dalhousie University Halifax NS, Canada, Department of Anesthesiology; [2]Department of Physiology and Biophysics Dalhousie University Halifax NS, Canada; [3]Department of Pathology Dalhousie University Halifax NS, Canada; [4]Department of Anesthesia, University of Toronto, ON, Canada; [5]Interdepartmental Division of Critical Care Medicine, University of Toronto; and Keenan Research Center at the Li Ka Shing Knowledge Institute of St. Michael's Hospital, Toronto ON, Canada; [6]Department of Surgical Sciences and Integrated Diagnostics, San Martino Policlinico Hospital - IRCCS for Oncology , University of Genoa, Italy; [7] Anesthesia and Surgical Intensive Care, Ruhr-University Bochum, Germany

## Supplementary Information

### Animal preparation

The University Committee on Laboratory Animals (UCLA), the Research Ethics Board, and the Carlton Animal Care Facility at Dalhousie University approved all experimental procedures. In an attempt to refine animal treatment in the laboratory, various measures were taken throughout experiments to ensure that the animals were treated with care and respect. All experiments were performed on a heated stainless steel operation table (Harvard Apparatus Canada, Saint-Laurent, QC, Canada) with an internal surface heater maintained at 37°C. Additional heat was also provided by an overhead lamp (Burton Medical, Chatsworth, CA, USA) as needed.

Sedation protocol: The animals were anesthetized with pentobarbital 55 mg/kg IP (Ceva Sante Animale, Montreal, QC, Canada) to provide surgical anesthesia for vessel cannulation. Once surgical anesthesia was confirmed, the animals were placed in supine position on the operation table, and the neck and femoral regions of the body were prepared for cut down and vessel cannulation. All animals received an intravenous infusion of remifentanil 4 µg/ml, diluted in saline (Ultiva®, Abbot Laboratories, QC, Canada) at a continuous rate of 0.4 µg /kg/min and intermittent doses of pentobarbital 0.25 mg IV every 30 min to maintain sedation and minimize pain and stress during mechanical ventilation. This dose was chosen based on physical assessments of the animals (toe pinch and tail flick), which had resulted in no responses from the animals to these stimuli in a previous methodological study (Ismaiel N, Chankalal R, Zhou J, Henzler D. Using remifentanil in mechanically ventilated rats to provide continuous analgosedation. J Am Assoc Lab Anim Sci. 2012;51(1):58-62). Hemodynamics, respiration and visual appearance were monitored continuously, while the level of sedation was additionally assessed every 30 min by the toe pinch and tail flick methods. In case of a reaction to stimuli or spontane-

ous movement a bolus of 0.2-0.4 μg/kg was allowed and additional pentobarbital 0.5 mg IV as rescue medication. All animals received 3-5 ml/h of NaCl 0.9% (Hospira, Montreal, QC, Canada) including occasional boluses as needed to flush the lines. Animals were kept supine throughout the protocol and were not restrained to notice any fleeing tendency due to inadequate sedation.

Instrumentation: A 14G endotracheal tube was inserted through a tracheostomy and animals were connected to a standard neonatal ventilator (EVITA4, Draeger Medical Canada Inc, Richmond, ON). 20G catheters were inserted into the common carotid artery and external jugular vein for monitoring of blood pressure. The femoral artery was cannulated with a thermocouple probe (ADInstruments Inc., Colorado Springs, CO) for cardiac output measurements.

Blood samples (0.3 ml) were analyzed for gas pressures and acid-base status and for oxygenation using a species adjusted hemoxymeter (ABL510 & OSM3, Radiometer Copenhagen, Denmark).

A fluid filled 20G catheter with multiple perforations was inserted into the esophagus for measurement of esophageal pressure and attached to a physiologic pressure transducer. The catheter was advanced into the stomach and slowly retracted until cardiac pulsations were minimal. Before each measurement, the catheter was flushed and re-zeroed to ensure patency.

**Figure S1**. Flow chart of experimental setup

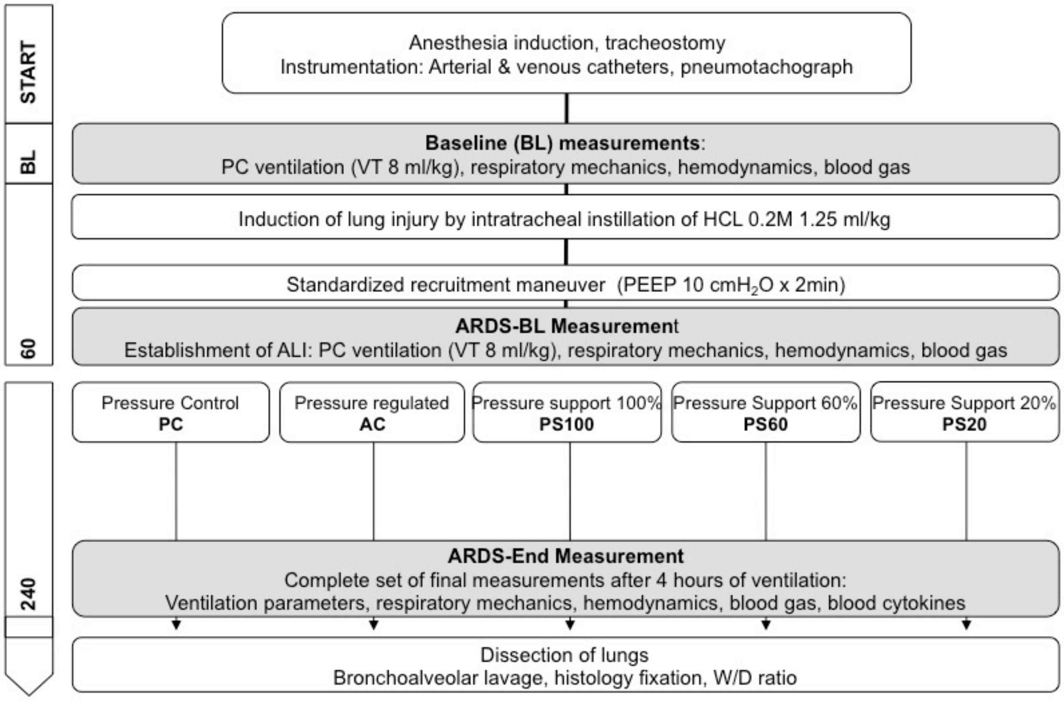

## Experimental protocol

Lung injury was induced by intra-tracheal instillation of 0.2m HCl as described previously[13]. Due to the different lung volumes, 0.3 ml were used for the right side and 0.2 ml for the left side. For each instillation, animals were positioned on the respective side and ventilated with 10-12 ml/kg BW. After 2 min the remainder was suctioned and the animal placed supine. Lungs were instilled sequentially allowing the animal to recover between instillations. After the manoeuvre animals were closely observed for any evidence of developing intrinsic PEEP (PEEPi). The expiratory flow curve was continuously monitored on the LabView system to assure end-expiratory zero flow. If needed, tracheal secretions were suctioned. Physiologic measurements were performed at baseline (BL) and 1h after induction of lung injury (ALI-BL). Interim measurements were taken every hour. The final measurement (ALI-End) was planned to take place 4 h after ALI-BL. If animals died prematurely, the last set of measurements before death was taken as ALI-End. Animals were only entered into the study and used for analysis, if they had received at least 120 min of the study ventilation after establishment of ALI. The times to measurements for all groups are given in table S1.

**Table S1.** Experimental time periods in min (mean ± SD)

| Group | Time BL to ALI-BL | Time BL to ALI-End measurement | | |
|---|---|---|---|---|
| | | All animals | Full length [n] | Premature death [n] |
| PC | 82 ± 18 | 256 ± 80 | 338 ± 11 [4] | 191 ± 34 [5] |
| AC | 81 ± 11 | 283 ± 58 | 315 ± 16 [7] | 210 ± 54 [3] |
| PS100 | 85 ± 15 | 293 ± 56 | 326 ± 22 [5] | 225 ± 28 [4] |
| PS60 | 84 ± 12 | 282 ± 51 | 320 ± 9 [6] | 225 ± 25 [4] |
| PS20 | 83 ± 22 | 240 ± 56 | 291 ± 40 [3] | 213 ± 38 [7] |

## Hemodynamic and respiratory measurements

Arterial pressures were directly transduced and recorded. Cardiac output was measured by transcardio-pulmonary temperature dilution of 0.5ml of saline solution (LabChart 6.0, ADInstruments). A data collection system (PowerLab, ADInstruments) was used for recording data and hemodynamic calculations

Gas flow and airway pressure (Paw) were measured proximal to the endotracheal tube by a differential pressure transducer from a heated pneumotachograph (PTN 113252, Hans Rudolph Inc., Shawnee, KS). Pressure and flow were zeroed and calibrated before each experiment using a modified manometer and a 20ml calibration syringe. The tidal volume ($V_T$) was calculated from integrating the flow signal. The minute ventilation ($V_E$) was calculated by multiplying the $V_T$ with the respiratory rate (RR).

The dynamic compliance ($C_{DYN}$) was defined as tidal volume ($V_T$) divided by the pressure difference between end-inspiratory and end-expiratory airway pressure. The maximum difference between inspiratory airway pressure and esophageal pressure ($P_{ES}$) was defined as peak inspiratory transpulmonary pressure ($P_{TPi}$). The pressure-time product (PTP) was calculated as the area under the baseline from the $P_{ES}$ curve.

The work of breathing (WOB) was calculated separately for total ($WOB_T$), ventilator ($WOB_V$) and respiratory muscles ($WOB_{RM}$) from the recorded flow and $P_{ES}$ tracings .

The work of breathing (WOB) was generally calculated as

$$\int P * f\, dt$$

where P is the driving pressure and f is the gas flow derived from application of that pressure. The $WOB_T$ was obtained by using transpulmonary pressure

$$\int P_{tp} * f\, dt$$

$WOB_V$ was derived using the $P_{AW}$ applied by the ventilator

$$\int P_{aw} * f\, dt$$

The work of breathing done by the subject was calculated by using $P_{ES}$ and the work needed to inflate the chest wall of a rat

$$\int P_{es} * f\, dt + W_{CW}$$

where $W_{CW} = \frac{{V_t}^2}{2\, C_w}$ with Cw = 1.5 ml/cm$H_2O$.
All calculations were done from automated extractions of data from the recorded curves that were entered manually into a spreadsheet (MS Excel). WOB are expressed in J/L of ventilation.

*References:* Lay YL, Hildebrandt J. Respiratory mechanics in the anesthetized rat. J Appl Physiol 1978; 45:255-60

## Histopathologic Examination

At the end of the experiment the animals were killed by a barbiturate overdose. The lungs were excised in inflated stated that was maintained with an inspiratory pressure of 10 cm$H_2O$. The left lung was ligated and fixated in inflated state with 10% buffered formalin for histopathologic examination. The right middle lobe was ligated, cut and weighted. It was dried for 48 h at 37°C and weighted again. The ratio wet-to-dry ratio was calculated. Broncho-alveolar lavage fluid (BALF) was obtained from repeated lavage of the right lung (3ml), snap frozen and stored at -80° for subsequent analysis.

After 48h of formalin fixation, lungs were embedded in paraffin, cut and stained with hematoxylin eosin (HE). A lung pathologist blinded to the experimental group (Z.X.) graded the injury using a modification of diffuse alveolar damage (DAD) scoring system. Three complete cross-sections (apical, middle and basal) were viewed with increasing magnification from 40x to 200x. First, the cross-sections were scanned, and then the areas of pathologic changes were characterized. Interstitial edema, alveolar edema, hemorrhage, neutrophil infiltration, atelectasis, hyaline membranes, and epithelial dam-

age/disruption were scored. 0-3 points were assigned to each parameter and the cumulative score calculated (0 = absent; 1 = mild, localized; 2 = moderate, large areas; 3 = severe, ubiquitous)(Table S2).

**Table S2**. Diffuse alveolar damage (DAD) score with components and grading scheme.

| Parameters | Scoring (for each) |
|---|---|
| Interstitial edema | 0 = absent |
| Alveolar edema | 1 = mild, localized |
| Hemorrhage | 2 = moderate, larger areas |
| Neutrophil infiltration | 3 = severe, ubiquitous |
| Atelectasis | |
| Hyaline membranes | Worst possible score: 21 |
| Alveolar epithelial damage | |

**Cytokine Analysis**

Blood samples were immediately centrifuged at 5000 rpm for 15 min and supernatant plasma snap frozen and stored at -80°. BALF and plasma samples were analyzed in duplicate by Luminex® technology on a 96-well (PC4110 multiplex kit, Panomics Inc., Fremont, CA) for: Tumor necrosis factor (TNFα), interleukin (IL)-1β, IL6, IL10, intracellular adhesion molecule (ICAM1), macrophage inflammatory protein (MIP1α), KC (CXCL1 chemokine), Regulated upon Activation Normal T-cell Expressed and Secreted (RANTES) and Monocyte Chemotactic Protein (MCP1). Standard dilution curves were constructed to calculate concentrations.

## Tables

**Table S3:** Work of breathing

| | | PC | AC | PS100 | PS60 | PS20 |
|---|---|---|---|---|---|---|
| $WOB_V$ | BL | 0.53 ± 0.27 | 0.46 ± 0.18 | 0.49 ± 0.22 | 0.44 ± 0.20 | 0.46 ± 0.18 |
| [J/l] | ALI End | 0.73 ± 0.47 [de] | 0.61 ± 0.57 [e] | 0.37 ± 0.33 | 0.15 ± 0.15* [a] | 0.05 ± 0.08* [ab] |
| $WOB_{RM}$ | BL | 0.05 ± 0.03 | 0.05 ± 0.04 | 0.04 ± 0.03 | 0.07 ± 0.04 | 0.06 ± 0.05 |
| [J/l] | ALI End | 0.08 ± 0.10 [cde] | 0.26 ± 0.24*[de] | 0.71 ± 0.41*[a] | 0.98 ± 0.38* [ab] | 1.08 ± 0.55* [ab] |
| $WOB_T$ | BL | 0.50 ± 0.26 | 0.44 ± 0.15 | 0.51 ± 0.23 | 0.39 ± 0.19 | 0.45 ± 0.17 |
| [J/l] | ALI End | 0.75 ± 0.55 | 0.84 ± 0.42* | 1.02 ± 0.53* | 1.0 ± 0.34* | 1.11 ± 0.53* |
| PTP | BL | 27.4 ± 22.8 | 26.1 ± 18.4 | 44.3 ± 40.9 | 41.3 ± 32.6 | 28.3 ± 20.8 |
| [$cmH_2O$*s*min$^{-1}$] | ALI End | 28.1 ± 45.3 [cde] | 139.1 ± 79.5* | 157.9 ± 144.1[a] | 188.8 ± 74.1* [a] | 204.1 ± 92.3*[a] |
| $MP_T$ | BL | 39.1 ± 24.5 | 35.1 ± 15.5 | 43.7 ± 20.0 | 33.6 ± 16.7 | 38.8 ± 16.3 |
| [J*l$^{-1}$*min$^{-1}$] | ALI End | 63.5 ± 41.9* | 70.2 ± 16.9* | 95.9 ± 70.2* | 92.5 ± 43.3* | 88.3 ± 45.7* |

$p < 0.05$ compared to: *= Baseline; [a]= CMV; [b]= A/C; [c]= PSV100; [d]= PSV60; [e]= PSV20. $WOB_V$: work of breathing performed by ventilator; $WOB_{RM}$: work of breatning performed by respiratory muscle; $WOB_T$: total work of breathing; $MP_T$: total mechanical power

**Table S4:** Lung damage. DAD subscores and total score (median[IQR])

| | PC | AC | PS100 | PS60 | PS20 |
|---|---|---|---|---|---|
| Edema interstitial | 2(1;2) | 1(0;2) | 2(1.75;2) | 1(0;2) | 1.5(1;2) |
| Edema alveolar | 1(1;2) | 2(1;2) | 1(1;2) | 1(1;1.25) | 1(1;1.25) |
| Hemorrhage | 1(0;1) | 1(0.75;1) | 1(1;1) | 1(0.75;2) | 1(0;1) |
| Neutrophil infiltration | 2(1;2.3) | 1(1;2.25) | 2(2;2) | 1(1;2) | 2(1;2) |
| Atelectasis | 2(0.75;2) | 1(0;2) | 1(0.75;2) | 0(0;1) | 1(0;1) |
| Hyaline membrane | 1(0;3) | 0(0;3) | 1(0;3) | 0.5(0;2) | 2(1;2) |
| Epithelial damage | 2(1;3) | 1(0;3) | 2(2;3) | 1.5(0.2) | 2(1.75;2) |
| Total DAD score | 12(4.75;14) | 7.5(4;13) | 11(9.75;12.5) | 8.5(3;9.5) | 9.5(8;10) |

**Table S5:** Cytokines BALF [pg/ml] (mean±SD)

| | PC | AC | PS100 | PS60 | PS20 |
|---|---|---|---|---|---|
| IL1b | 8.2±2.9 | 9.4±2.8 | 10.7±3.5 | 8.8±1.3 | 8.7±3.6 |
| ICAM x 1000 | 8.8±6.1 | 10.9±5.8 | 9.8±3.7 | 7.0±4.3 | 8.0±4.9 |
| IL6 | 462±151 | 516±216 | 489±161 | 387±172 | 427±133 |
| TNFa | 26.2±13.8 | 39.6±21.2 | 34.5±10.1 | 28.8±13.2 | 32.8±11.2 |
| IL10 | 154±155 | 205±135 | 285±517 | 81±56 | 119±134 |
| GMCSF | 0.7±1.3 | 1.1±1.7 | 0.8±1.9 | 0±0 | 1.2±2.5 |
| KC | 171±149 | 204±123 | 261±166 | 287±224 | 419±328 |
| RANTES | 23.1±7.2 | 31.2±6.6 | 30.2±6.1 | 24.5±6.0 | 27.4±6.2 |
| MIP1a | 39.4±8.7 | 43.7±14.2 | 51.0±30.0 | 46.2±22.2 | 49.4±18.4 |
| MCP1 x1000 | 11.7±8.8 | 14.4±5.2 | 11.4±6.2 | 9.1±4.8 | 8.0±2.8 |

**Table S6:** Cytokines Blood – arterial [pg/ml] (mean±SD)

| | PC | AC | PS100 | PS60 | PS20 |
|---|---|---|---|---|---|
| IL1b | 9.1±3.6 | 5.8±2.2 | 13.0±11.2 | 10.6±4.5 | 63.5±119 |
| ICAM x1000 | 8.2±4.7 | 9.2±5.7 | 10.4±5.1 | 9.4±2.6 | 7.8±2.3 |
| IL6 | 46.7±36.4$^{\#}$ | 78.4±58.9$^{\#}$ | 36±18.8$^{\#}$ | 32±10.8$^{\#}$ | 44.8±35.6$^{\#}$ |
| TNFa | 39.7±35.7 | 36.5±35.5 | 32.4±36.9 | 31±8.4 | 29.5±12.1 |
| IL10 | 235±150 | 218±181 | 177±103 | 226±115* | 341±408* |
| GMCSF | 3.4±3.9 | 3.8±2.9 | 5.1±3.8 | 6.5±4.1 | 50.5±108.9 |
| KC | 9.4±10.5$^{\#}$ | 13.3±13.1$^{\#}$ | 16±31.2$^{\#}$ | 7.1±3.2$^{\#}$ | 8.9±5.8$^{\#}$ |
| RANTES | 37.0±20.8* | 45.7±47.9* | 36.9±10.6* | 44.1±10.9* | 54.3±18.5* |
| MIP1a | 32.1±24.2$^{\#}$ | 31.4±19.3$^{\#}$ | 36.4±17.3$^{\#}$ | 49.4±24.9$^{\#}$ | 28.4±9.5$^{\#}$ |
| MCP1 x1000 | 0.74±0.63$^{\#}$ | 0.84±0.46$^{\#}$ | 0.81±0.61$^{\#}$ | 0.69±0.43$^{\#}$ | 0.95±0.81$^{\#}$ |

* higher than BALF ($p<0.05$); # lower than BALF ($p<0.05$)

**Table S7:** Cytokines Blood – venous [pg/ml] (mean±SD)

| | PC | AC | PS100 | PS60 | PS20 |
|---|---|---|---|---|---|
| IL1b | 6.4±3.4 | 5.1±1.6 | 11.1±5.8 | 9.1±1.7 | 40.3±64.1 |
| ICAM x1000 | 8.6±5.2 | 11.5±5.9 | 8.4±5.0 | 8.6±2.5 | 8.4±2.9 |
| IL6 | 44.2±40.8 | 77.3±66.3 | 43.3±31.4 | 29.5±12.1 | 40.1±32.2 |
| TNFa | 33.6±20.2 | 30±17.9 | 35±36.2 | 29.5±13.9 | 34±13.3 |
| IL10 | 225±162 | 232±210 | 228±162 | 220±130 | 373±405 |
| GMCSF | 2.5±3.1 | 2.8±3.0 | 5.5±4.9 | 4.9±3.6 | 45.6±101.4 |
| KC | 9.3±9.3 | 18±19.8 | 21.9±49.4 | 5.7±3.8 | 12.5±11.9 |
| RANTES | 35.8±19.3 | 41.1±20.4 | 38.5±16.9 | 38.6±8.3 | 48.9±12.8 |
| MIP1a | 22.3±11 | 29.6±15.1 | 32.7±18.3 | 43.2±27.3 | 28.2±8.5 |
| MCP1 x1000 | 0.77±0.52 | 1.11±0.43 | 0.65±0.65 | 0.79±0.51 | 0.96±0.61 |

## Figures

**Figure S2**. Experimental design. Original tracings of flow, airway, esophageal and transpulmonary pressure.

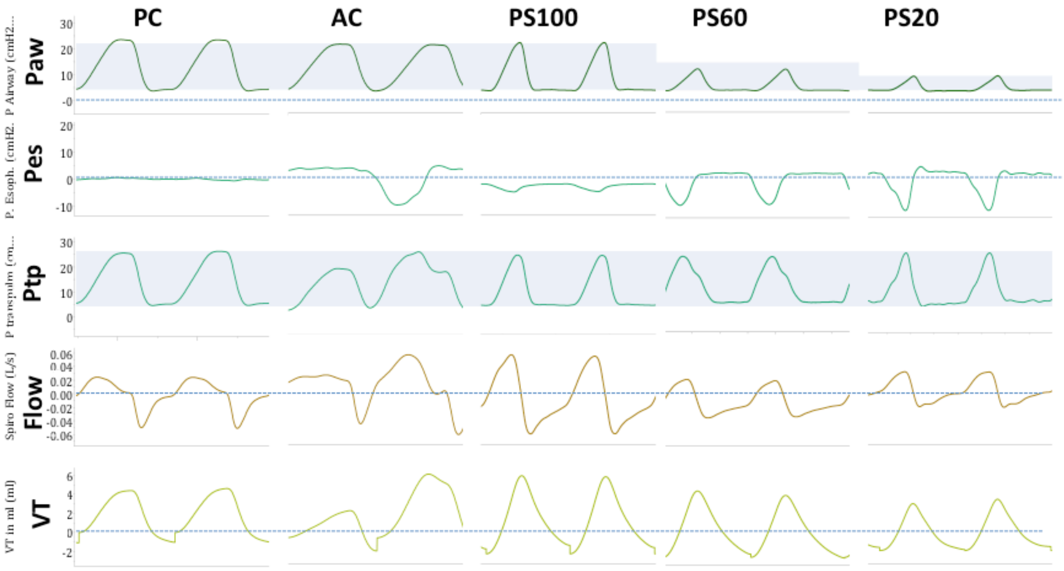


PC pressure-control; AC assist-control; PS pressure support ventilation with 100%(PS100), 60%(PS60) or 20%(PS20) of previous pressure control level.

**Figure S3:** Plot of mean arterial pressure vs. work of breathing. The more of the work performed by the ventilator, the lower the arterial pressure (r=-0.389, p=0.007)

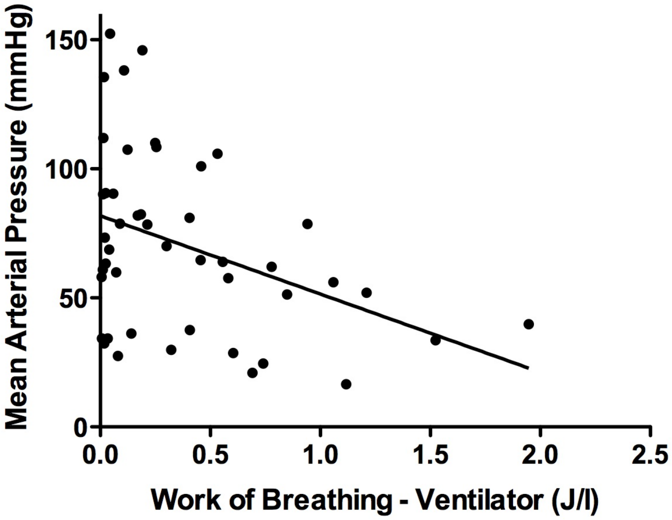

**Figure S4**. Wet-to-dry ratio (W/D) of rat lung. Boxplot.

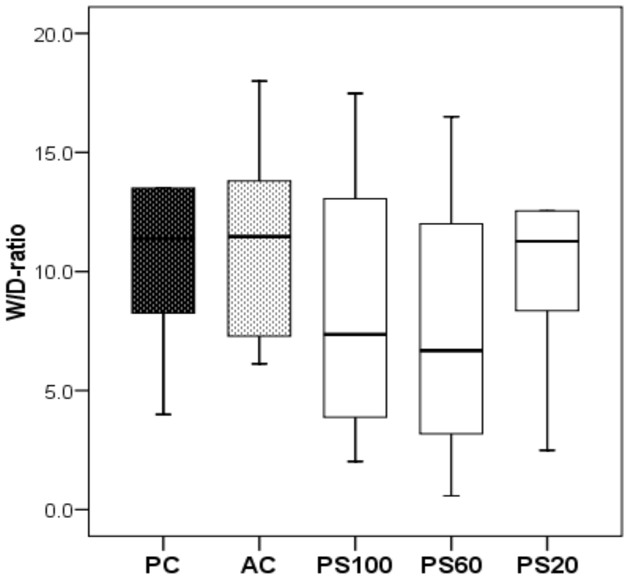


Groups are pressure-control (PC), assist-control (AC), or pressure support ventilation with 100%(PS100), 60%(PS60) or 20%(PS20) of previous pressure control level

**Figure S5.** Correlation of pressure-time product (PTP) with mean diffuse alveolar damage score (DAD). r = -0.469, p = 0.049.

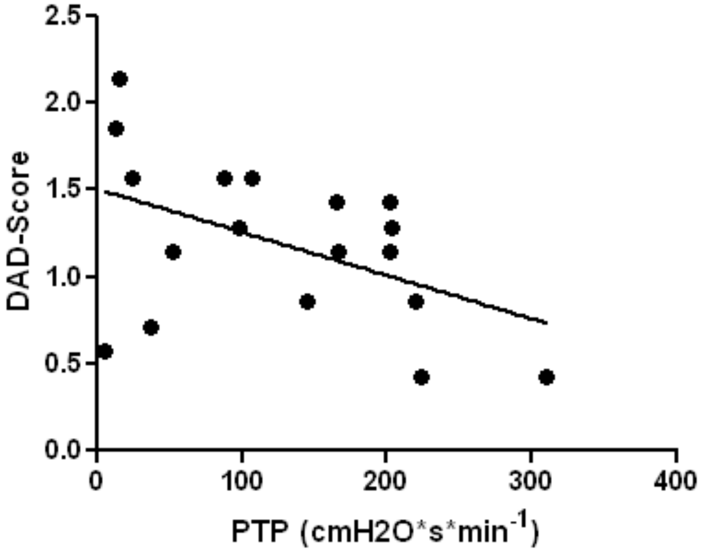

**Figure S6**. Histology slides. H&E staining, magnification x40

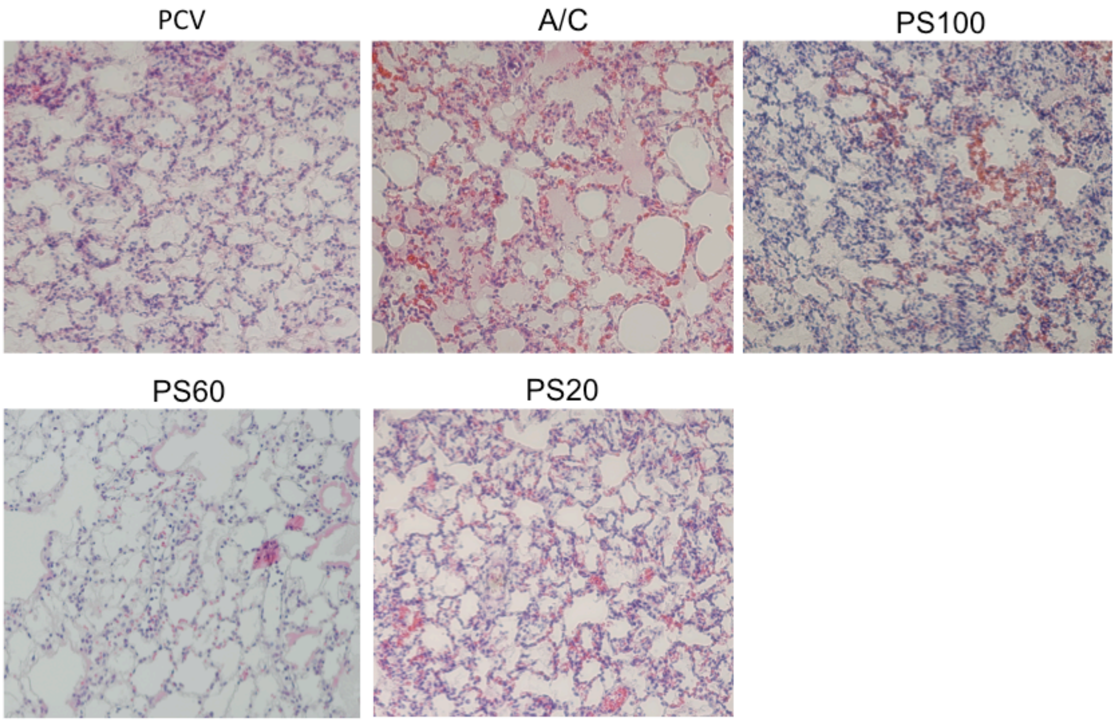

Supplement: Supplementary file 1 — Additional file 1: Supplemental digital content [file 40635_2019_272_MOESM1_ESM.pdf]
